# Impact of Fermented or Enzymatically Fermented Dried Olive Pomace on Growth, Expression of Digestive Enzyme and Glucose Transporter Genes, Oxidative Stability of Frozen Meat, and Economic Efficiency of Broiler Chickens

**DOI:** 10.3389/fvets.2021.644325

**Published:** 2021-05-28

**Authors:** Doaa Ibrahim, Amira Moustafa, Sara E. Shahin, Wafaa R. I. A. Sherief, Karima Abdallah, Mohamed F. M. Farag, Mohamed A. Nassan, Seham M. Ibrahim

**Affiliations:** ^1^Department of Nutrition and Clinical Nutrition, Faculty of Veterinary Medicine, Zagazig University, Zagazig, Egypt; ^2^Department of Physiology, Faculty of Veterinary Medicine, Zagazig University, Zagazig, Egypt; ^3^Department of Animal Wealth Development, Veterinary Economics and Farm Management, Faculty of Veterinary Medicine, Zagazig University, Zagazig, Egypt; ^4^Department of Animal Wealth Development, Animal Breeding, and Production, Faculty of Veterinary Medicine, Zagazig University, Zagazig, Egypt; ^5^Food Control Department, Faculty of Veterinary Medicine, Zagazig University, Zagazig, Egypt; ^6^Department of Clinical Pathology, Faculty of Veterinary Medicine, Zagazig University, Zagazig, Egypt; ^7^Department of Clinical Laboratory sciences, Turabah University College, Taif University, Taif, Saudi Arabia

**Keywords:** olive pomace, growth, gene expression, profitability, meat quality

## Abstract

The use of dried olive pomace as complementary energy sources in poultry feed is still limited due to its low protein and high fiber contents. Bioconversion of olive pomace through solid-state fermentation with or without exogenous enzymes is considered as a trial for improving its nutritional value. This study aimed to evaluate the effects of fermented olive pomace with or without enzymatic treatment on the growth, modulations of genes encoding digestive enzymes and glucose transporters, meat oxidative stability, and economic efficiency of broiler chickens. A total of 1400 day-old broiler chicks (Ross 308) were randomly allocated to seven dietary treatments with 10 replicates of 20 birds/replicate. Treatments included control (basal corn–soybean diet) and other six treatments in which basal diet was replaced by three levels (7.5, 15, and 30%) of fermented olive pomace (FOPI) or enzymatically fermented olive pomace (FOPII) for 42 days. The highest body weight gain was observed in groups fed 7.5 and 15% FOPII (increased by 6.6 and 12.5%, respectively, when compared with the control group). Also, feeding on 7.5 and 15% FOPII yielded a better feed conversion ratio and improved the digestibility of crude protein, fat, and crude fiber. The expression of the *SGLT-1* gene was upregulated in groups fed FOPI and FOPII when compared with the control group. Moreover, the expression of the *GLUT2* gene was elevated in groups fed 7.5 and 15% FOPII. By increasing the levels of FOPI and FOPII in diets, the expression of genes encoding pancreatic *AMY2A, PNLIP*, and *CCK* was upregulated (*p* < 0.05) when compared with the control. Fat percentage and cholesterol content in breast meat were significantly reduced (*p* < 0.05) by nearly 13.7 and 16.7% in groups fed FOPI and FOPII at the levels of 15 and 30%. Total phenolic and flavonoid contents in breast meat were significantly increased in groups fed 15 and 30% FOPI and FOPII when compared with the control group and even after a long period of frozen storage. After 180 days of frozen storage, the inclusion of high levels of FOP significantly increased (*p* < 0.05) the levels of glutathione peroxide and total superoxide dismutase and meat ability to scavenge free radical 1,1-diphenyl-2-picrylhydrazyl. Furthermore, the highest net profit and profitability ratio and the lowest cost feed/kg body gain were achieved in groups fed 7.5 and 15% of FOPII, respectively. The results of this study indicated that dietary inclusion of 15% FOPII could enhance the growth performance and economic efficiency of broiler chickens. Moreover, a higher inclusion level of FOPI or FOPII could enhance the quality and increase the oxidative stability of frozen meat and extend the storage time.

## Introduction

Agro-industrial by-products can provide an alternative feed source for livestock which offer an eco-friendly approach for their disposal and recycling. In the past, utilization of crop residues and by-products (such as brewers dried grain and olive pomace) as alternatives to corn in poultry feed was not successful, mainly due to their high fiber content and poor digestibility (1–3). Additionally, the quality of poultry meat is strongly related to an animal diet; thus, modulation of the animal feed could account for higher quality and nutritional value of their products (4, 5). Olive pomace (OP), the solid by-product generated from olive oil processing, is considered as a good source of functional compounds (simple phenolics, polyphenols, oleuropeoside, and flavonoid) that enhance animal health and performance (6). Olive pomace is relatively rich in water, contains high fiber content (lignin, cellulose, hemicellulose, and pectin) (7), and is rich in fat, mainly polyunsaturated fatty acids (PUFAs) (8, 9). Recently, the inclusion of dried olive pomace (DOP) in the feeding of broiler chickens has attracted special attention for the following reasons. Firstly, DOP is considered as a low-cost complementary energy source (10) due to its high oil content. Secondly, it has a higher amount of PUFAs, which account for meat fatty acid composition (11). Thirdly, DOP can be considered as an excellent source of natural antioxidants (12, 13), such as oleuropein and hydroxytyrosol (14), which help in impeding the oxidative consequences in the muscle tissues. Some studies described that inclusion of dietary dried olive pulp up to 10% had no negative effects on growth performance of broilers (15–17). Moreover, broiler chickens can utilize dried olive pulp more effectively with increasing age (18). This can be attributed to the presence of high fiber content comprising non-starch polysaccharides (16) that limited its use in broilers' diet especially at their early age with immature digestive tract (19). Several feeding practices have been established for increasing olive pomace utilization aiming to produce economic value-added by-products with the reduction of environmental-polluting load (20). Among these new strategies, fermentation, which is a dynamic process, uses microorganisms, substrates, and environmental conditions to transform complicated substrates into simpler form (21). Additionally, the application of probiotic fermentation technologies can increase the concentration of metabolites, enzymes, and probiotics in the feed (22). Fermented feed application in chickens' diets could improve their nutritional properties through decreasing crude fiber and increasing crude protein contents (23, 24), eliminating numerous antinutritional factors and toxic components in feed ingredients (25). Moreover, supplementation of exogenous microbial enzymes at the time of feeding has the benefit of solubilizing phytic phosphorus (phytase) and facilitating the digestion of fiber (carbohydrases) in poultry diets (26). However, the optimum efficiency of these enzymes is limited when used directly in feed, due to limited time of feed retention in the gastrointestinal tract (GIT) of chickens, and since their optimal pH is between 4 and 6, the degradation activity of these enzymes is mostly limited to crop, proventriculus, and gizzard (27, 28). Thus, the application of these enzymes on feed ingredients prior to the time of feeding may have an additional benefit on birds' performance more than its application at the time of feeding. Moreover, using these enzymes especially in the presence of microbial inoculants has been shown to enhance the fermentation process (29) and account for more nutritional value of fermented products. Besides improving the nutritional value of fermented feed, fermentation has been demonstrated to enhance the digestibility of several nutrients such as dry matter, crude protein, and crude fiber (30, 31). Additionally, it increases the palatability of feed (32, 33), improves growth performance, and enhances beneficial gut microbiota and immune resistance in broiler chickens (34). The nature of broiler's diet (35) can influence the digestion and absorption of dietary nutrients by regulating digestive enzymes and transporter proteins in intestinal enterocytes (2, 36). Therefore, evaluating the expression of genes encoding digestive enzymes (pancreatic amylase, lipase, and cholecystokinin) and sugar transporters (glucose transporter 2, GLUT2; and sodium/glucose cotransporter 1, SGLT1) after feeding on fermented olive pomace may reflect its ability to improve broiler's growth performance.

Although using olive pomace has many benefits in the poultry diet, it also has some limitations which may be overcome by fermentation technology with the addition of exogenous enzymes. The present study was conducted to investigate the effect/s of fermented olive pomace on broiler's growth performance by modulating the expression of digestive enzymes (lipase, amylase, and cholecystokinin) and glucose transporter genes and the economic efficiency and meat quality offered for human consumption.

## Materials and Methods

All the experimental processes were done at the Institute of Nutrition and Clinical Nutrition and Poultry Farm following the Faculty of Veterinary Medicine guidelines, and the protocols were approved by the Institutional Animal Care and Use Committee of Zagazig University.

### Two-Stage Solid Fermentation of Olive Pomace Without Enzymatic Treatment

In the preparation of olive pomace, OP was obtained from a local olive-pressing factory and then anti-mycotoxin (Mycofix, Biomin) was added. In the next step, OP was dried at 70°C using hot air and sieved (1.5 mm mesh diameter) to remove part of the stones (seeds). *Bacillus subtilis* var. *natto* N21 (BS) and *Lactobacillus casei* were used for dried olive pomace fermentation.

For microbial activation, tryptone soya broth (BD) was used for the incubation of BS at 37°C in a 150-rpm Erlenmeyer flask. Lactobacilli MRS broth (BD) was used for the incubation of *L. casei* at 37°C in a 100-rpm Erlenmeyer flask. After that, the broth was centrifuged for 10 min, the supernatant was discarded, and sterile water was added to obtain 10^9^ CFU/mL. For the preparation of fermented feed, *B. subtilis* was added at a concentration of 10^6^ CFU/g of feed and with 10% water for 2 days of aerobic fermentation at 37°C in the first phase, and then LC was added (10^6^ CFU/g feed) with 13% water for 5 days of anaerobic fermentation at 25 to 35°C in the second phase.

### Two-Stage Solid Fermentation of Olive Pomace With Enzymatic Treatment

The microbial fermentation of olive pomace in this trial was done as previously described by *B. subtilis* var. *natto* N21 and *L. casei*; besides, commercial exogenous enzymes (HOSTAZYME-X, Huvepharma, Inc. 525 Westpark Drive, Suite 230, Peachtree City, GA 30269, USA) consisting of beta xylanase–beta-glucanase were added at the starting point of fermentation at the level 50 g/ton olive pomace.

### Drying and Processing of Fermented Olive Pomace With and Without Enzymatic Treatment

The fermented feed was dried using an oven and mixed with feed ingredients. The feed moisture content was below 12%. Chemical analysis of unfermented and fermented OP with and without enzymatic treatment was done as described by AOAC (37) (Table 1). Acid detergent fiber (ADF), neutral detergent fiber (NDF), and ash-free NDF were estimated according to Van Soest et al. (38). Hemicellulose was calculated as the difference between NDF and ADF, whereas cellulose was the difference between the ADF and ADL method. pH value was estimated by a digital pH meter (Hanna HI-2211). For counting total lactic acid bacteria, sterile water (9 ml) was added to DOP (1 g) before and after fermentation and then mixed. Buffered peptone water was used to dilute the supernatants (10-fold). Lactobacilli De Man and Rogosa and Sharpe agar (MRS, CM1153, Oxoid) were added to 100 μm of supernatant and incubated for 48 h at 37°C with 13% CO_2_.

**Table 1:** Chemical analysis of unfermented olive pomace (UFOP), fermented olive pomace (FOPI), and fermented olive pomace with exogenous enzymes (FOPII).

**Parameters**	**UFOP**	**FOPI**	**FOPII**	***p*-value**	**SEM**
Organic matter, %	92.73^b^	92.27^c^	93.60^a^	<0.04	0.11
Crude protein, %	10.34^c^	11.20^b^	12.92^a^	<0.02	0.21
Crude fiber, %	30.5^a^	22.67^b^	20.33^c^	<0.001	0.84
Ether extract, %	14.87^c^	15.90^b^	17.87^a^	<0.001	0.25
Lignin, %	8.53^a^	7.10^b^	6.60^c^	<0.001	0.16
Cellulose, %	40.20^a^	33.30^b^	30.23^c^	<0.001	0.81
Hemicellulose, %	8.27^c^	11.30^b^	13.43^a^	<0.005	0.41
Neutral detergent fiber (NDF), %	57.00^a^	51.70^b^	50.27^c^	<0.001	0.56
Acid detergent fiber (ADF), %	48.73^a^	40.4^b^	36.83^c^	<0.001	0.97
Total polyphenols (g/kg dry matter)	2.37^a^	4.17^b^	4.08^b^	<0.001	0.16
pH of starter diet	6.43^a^	4.66^b^	4.25^c^	<0.008	0.18
pH grower–finisher diet	6.35^a^	4.28^b^	4.17^c^	<0.03	0.19
Total lactic bacteria (log CFU/g feed)	4.16^c^	8.17^b^	9.05^a^	<0.001	0.41

*Values are expressed as means ± standard error*.

*Means within the same row carrying different superscripts (^a−c^) are significantly different at p < 0.05*.

### Birds, Experimental Design, and Diet

Seven hundred, 1-day-old, male broiler chicks (Ross 308) were separately weighed and distributed to seven dietary treatments, each treatment comprising 10 replicates of 20 chicks each per floor pen and each pen considered as an experimental unit. Dietary treatment included the following: control (basal corn–soybean diet) and other six treatments in which basal diet was replaced by three levels (7.5, 15, and 30%) of FOPI or FOPII. A two-phase feeding program was applied that included a starter phase (days 1–21) and a finisher phase (days 22–42). The birds were fed a basal diet formulated according to Ross broiler nutrition specification (Tables 2, 3). All chicks were given *ad libitum* access to feed and water. The temperature, lighting, and relative humidity were controlled according to the guidelines of Ross 308 management (39). The proximate analysis of feed ingredients was done according to the standard method of the AOAC (37). All experimental diets were offered in mash following the nutrition specification of the Ross broiler handbook (39). The total phenolic contents (TPC) in olive pomace samples before and after fermentation were measured by adopting the procedure as described by Seneviratne et al. (40). The TPC were estimated as g/kg dry matter.

**Table 2:** The ingredients and nutrient level of diets during the starter stage.

**Ingredients**	**Control**	**FOPI7.5%**	**FOPI15%**	**FOPI30%**	**FOPII7.5%**	**FOPII15%**	**FOPII30%**
Yellow corn	57.75	50.50	43.30	28.70	51.00	44.30	31.00
Soybean meal, 46%	32.30	31.60	31.00	29.75	31.30	30.30	28.20
Corn gluten	3.60	3.60	3.60	3.60	3.60	3.60	3.60
FOP	-	7.50	15.00	30.00	7.5.00	15.00	30.00
Soybean oil	2.50	2.90	3.20	4.00	2.7	2.9	3.20
Calcium carbonate	1.10	1.10	1.10	1.10	1.10	1.10	1.10
Calcium diphasic phosphate	1.10	1.10	1.10	1.10	1.10	1.10	1.10
Common salt	0.30	0.30	0.30	0.30	0.30	0.30	0.30
Premix^a^	0.50	0.50	0.50	0.50	0.50	0.50	0.50
l-Lysine	0.40	0.45	0.45	0.50	0.45	0.45	0.55
dl-Methionine	0.15	0.15	0.15	0.15	0.15	0.15	0.15
Choline chloride	0.20	0.20	0.20	0.20	0.20	0.20	0.20
Anti-mycotoxin	0.10	0.10	0.10	0.10	0.10	0.10	0.10
Analyzed composition^b^						
ME (kcal/kg)	3,100	3,103	3,100	3,101	3,101	3,101	3,100
CP (%)	22.52	22.54	22.52	22.53	22.55	22.51	22.50
EE %	4.93	6.20	7.53	10.19	6.15	7.43	10.12
CF (%)	2.54	4.00	5.38	7.33	3.86	5.21	6.26
Calcium (%)	0.93	0.92	0.99	1.12	1.10	1.08	1.08
Available phosphorous (%)	0.42	0.43	0.45	0.44	0.44	0.42	0.44
Lysine (%)	1.44	1.45	1.44	1.43	1.45	1.42	1.44
Methionine (%)	0.54	0.51	0.50	0.50	0.54	0.50	0.50

*FOPI7.5%, 7.5% olive pomace subjected to microbial fermentation; FOPI15%, 15% olive pomace subjected to microbial fermentation; FOPI30%, 30% olive pomace subjected to microbial fermentation; FOPII7.5%, 7.5% olive pomace subjected to microbial and enzymatic fermentation; FOPII15%, 15% olive pomace subjected to microbial and enzymatic fermentation; FOPII30%, 30% olive pomace subjected to microbial and enzymatic fermentation*.

a *Vitamin premix supplied per kilogram of diet: vitamin A, 10,000 IU; vitamin D_3_, 2,000 IU; vitamin E, 6,500 IU; vitamin K_3_, 1 mg; vitamin B_1_, 2,560 mg; vitamin B_2_, 5,000 mg; vitamin B_6_, 1,500 mg; B_5_, 8 mg; niacin, 20,000 mg; biotin, 0.25 mg; folic acid, 1,000 mg; vitamin B_12_, 60 mg; Cu, 8 mg; Fe, 80 mg; Mn, 60 mg; Zn, 40 mg; Se, 0.15 mg*.

b *Calculated values for metabolizable energy and amino acids*.

**Table 3:** The ingredients and nutrient level of diets during the grower–finisher stage.

**Ingredients**	**Control**	**FOPI7.5%**	**FOPI15%**	**FOPI30%**	**FOPII7.5%**	**FOPII15%**	**FOPII30%**
Yellow corn	63.20	56.00	48.60	34.00	56.55	49.75	36.2
Soybean meal, 46%	25.60	25.00	24.50	23.35	24.50	23.6	21.80
Corn gluten	4.80	4.80	4.80	4.80	4.80	4.80	4.80
FOP	–	7.50	15.00	30.00	7.50	15.00	30.00
Soybean oil	3.00	3.30	3.70	4.40	3.20	3.4	3.70
Calcium carbonate	0.50	0.50	0.50	0.50	0.50	0.50	0.50
Calcium diphasic phosphate	1.30	1.30	1.30	1.30	1.30	1.30	1.30
Common salt	0.30	0.30	0.30	0.3	0.30	0.30	0.30
Premix^a^	0.50	0.50	0.50	0.5	0.50	0.50	0.50
l-Lysine	0.35	0.35	0.35	0.4	0.40	0.40	0.45
dl-Methionine	0.15	0.15	0.15	0.15	0.15	0.15	0.15
Choline chloride	0.10	0.10	0.10	0.20	0.20	0.20	0.20
Anti-mycotoxin	0.10	0.20	0.20	0.10	0.10	0.10	0.10
Analyzed composition^b^							
ME (kcal/kg)	3,206	3,201	3,202	3,200	3,207	3,206	3,200
CP (%)	20.45	20.48	20.47	20.45	20.53	20.43	20.52
EE %	5.60	6.22	8.10	10.40	6.85	8.12	10.60
CF (%)	2.42	3.93	5.45	7.40	3.78	5.10	6.65
Calcium (%)	0.79	0.80	0.82	0.83	0.80	0.84	0.82
Available phosphorous (%)	0.41	0.42	0.41	0.44	0.40	0.41	0.39
Lysine (%)	1.23	1.21	1.20	1.20	1.24	1.22	1.21
Methionine (%)	0.51	0.50	0.48	0.45	0.49	0.48	0.49

*FOPI7.5%, 7.5% olive pomace subjected to microbial fermentation; FOPI15%, 15% olive pomace subjected to microbial fermentation; FOPI30%, 30% olive pomace subjected to microbial fermentation; FOPII7.5%, 7.5% olive pomace subjected to microbial and enzymatic fermentation; FOPII15%, 15% olive pomace subjected to microbial and enzymatic fermentation; FOPII30%, 30% olive pomace subjected to microbial and enzymatic fermentation; CP, crude protein; EE, ether extract; CF, crude fiber*.

a *Vitamin premix supplied per kilogram of diet: vitamin A, 10,000 IU; vitamin D_3_, 2,000 IU; vitamin E, 6,500 IU; vitamin K_3_, 1 mg; vitamin B_1_, 2,560 mg; vitamin B_2_, 5,000 mg; vitamin B_6_, 1,500 mg; B_5_, 8 mg; niacin, 20,000 mg; biotin, 0.25 mg; folic acid, 1,000 mg; vitamin B_12_, 60 mg; Cu, 8 mg; Fe, 80 mg; Mn, 60 mg; Zn, 40 mg; Se, 0.15 mg*.

b *Calculated values for metabolizable energy and amino acids*.

### Growth Performance and Nutrient Digestibility

Average body weight (BW) and feed intake were estimated during the grower and finisher period, then BW gains and feed conversion ratio (FCR) were calculated for each phase and for the total growing period over 42 days. Total protein intake was calculated and then protein efficiency ratio (PER) was calculated as weight gain (g)/protein intake (g).

For apparent digestibility of nutrient estimation, 3 g TiO_2_ (as an indigestible marker) was added to each experimental diet. The excreta of chickens were collected for 7 days with the removal of any contamination and then dried at 65°C for 72 h and stored at −20°C. The TiO_2_ content in the excreta and diet was calculated after acid digestion. The calculation was done as follows: Apparent nutrient digestibility = 100 – [100 × (Indicator content (diet) / Indicator content (feces) × Nutrient content (feces) / Nutrient content (diet)].

### Sampling and Analytical Procedures

At day 42 of age, the birds were randomly selected and euthanized by cervical dislocation and the blood samples (*n* = 5/replicate) were collected for separation of the serum and stored at −20°C until further biochemical analyses. After blood collection, birds were defeathered and eviscerated and abdominal fat was collected. Samples (*n* = 5/replicate) from breast meat were immediately harvested and stored at −20°C until analysis of moisture, protein, fat, and cholesterol content and antioxidant capacity. Small pieces (*n* = 5/replicate) from the pancreas and duodenum were removed, flushed with phosphate buffer saline, and stored at −80°C in an Eppendorf cap lock tube for further RNA extraction.

### Organoleptic Examination of Chicken Breast

Organoleptic examination evaluated chicken breast samples for their color, odor, taste, and consistency according to the method recommended by Escobedo del Bosque et al. (41). Pinkish white color, fresh fleshy odor, palatable taste, and firm in consistency described a normal examination. Meanwhile, grayish color, rancid odor, unpalatable taste, and softness and slimness inconsistency described abnormal examination.

### Chemical Composition and Cholesterol Content in Breast Meat

The moisture protein and fat content of breast samples were determined following AOAC (37). The total cholesterol content in breast meat was estimated by gas chromatography, as previously described by Allain et al. (42).

### Biochemical Indices in Serum and Breast Meat

Serum total cholesterol, triglyceride, and low-density lipoprotein-cholesterol (LDL-C) concentrations were determined colorimetrically with a spectrophotometer using triglyceride (TR0100), total cholesterol (MAK043), and LDL/VLDL (MAK045) kits from Sigma Aldrich, following the manufacturer's instructions. Serum and meat filtrates were used for measuring the activity of the glutathione peroxidase (GSH-Px) using a commercial assay kit (Sigma-Aldrich, G6137). Total antioxidant capacity (T-AOC) was determined using a commercial assay kit (Sigma-Aldrich, MAK187), and the activity of superoxide dismutase (SOD) enzyme was determined using a commercial assay kit (Sigma-Aldrich, 19160) following the instructions of the test kit.

### Antioxidant Potential of Meat

#### Free Radical Scavenging Activity DPPH Assay

The free radical scavenging activity of the breast meat samples was examined by 1,1-diphenyl-2-picrylhydrazyl radical (DPPH) according to Jang et al. (43). Briefly, breast meat samples were homogenized and centrifuged. The supernatant was collected and mixed with DPPH radical solution and ethanol and then incubated for 10 min in a dark room. The absorbance measurement was read at 517 nm. The scavenging capacity for the DPPH radical was expressed as μM/g of wet muscle tissue.

#### Thiobarbituric Acid-Reactive Assay

Lipid oxidation was assessed 3 and 6 months from storage by the thiobarbituric acid method (44). Meat samples (5 g) were homogenized and the thiobarbituric acid-reactive assay (TBARS) value was measured as described by Ahn et al. (44). Briefly, ml TBA/TCA (trichloroacetic acid) solution (20 mM TBA in 15% TCA) and 50 μl butylated hydroxyanisole were mixed in the test tube. Tubes were heated for 30 min at 90°C in a boiling water bath, cooled, and then centrifuged for 15 min. The absorbance of the supernatant was determined at 532 nm with a spectrophotometer. TBARS value was expressed as mg malondialdehyde (MDA)/kg meat.

#### Total Phenolic Contents

TPC in breast broiler meat samples were determined according to the procedure described by Senevirathne et al. (45). Ethanol (500 μl, 95%), distilled water (2.5 ml), and Folin–Ciocalteu reagent (250 μl, 50%) were added to the homogenized meat sample (100 μl). After 5 min, Na_2_CO_3_ (250 μl, 5%) was added to the resultant mix, vortexed, and kept for 1 h in a dark room. Subsequently, the absorbance of the samples was determined at 725 nm *via* a spectrophotometer. The quantity of total phenolic compounds in breast meat samples was measured as gallic acid equivalent (mg gallic acid/100 g meat).

#### Determination of Total Flavonoids

Total flavonoid content was measured according to Meda et al. (46). In brief, 0.25 ml of the sample was mixed with 1 ml of double-distilled water. Then, 0.075 ml of NaNO_2_, 0.075 ml of 10% AlCl_3_, and 0.5 ml of 1 M NaOH were added in order. After that, the volume of the reacting solution was adjusted with double-distilled water to 2.5 ml. The absorbance of the solution was detected using the UV–visible spectrophotometer at a wavelength of 410 nm. For quantification of total flavonoid content, quercetin was used as standard. Results were expressed in microgram quercetin equivalents (QE)/mg.

### RNA Extraction and Reverse-Transcription Polymerase Chain Reaction

RNA isolation was done using a protocol of QIAamp RNeasy Mini kit (Qiagen GmbH, Hilden, Germany). Pancreatic and duodenal samples were frozen in liquid nitrogen in Eppendorf. RNA concentration was tested by Spectrostar NanoDrop^TM^ 2000 spectrophotometer (Thermo Fisher Scientific Inc., Waltham, MA, USA) at an optical density of 260 nm.

For SYBR green RT-PCR, the amplification of PCR was achieved in 25-μl reactions containing 0.25 μl of RevertAid reverse transcriptase (Thermo Fisher Scientific, Germany), 12.5 μl of 2× QuantiTect SYBR Green PCR master mix (Qiagen), 0.5 μl of each primer, 8.25 μl of RNase-free water, and 3 μl of the RNA template. The amplification of real-time PCR was carried out using a Rotor-Gene Q2 plex (Qiagen Inc., Valencia, CA, USA). The primers' sequences of pancreatic alpha 2A amylase genes (*AMY2A*), lipase (*PNLIP*), and cholecystokinin (*CCK*) and glucose transporter-2 (*SLC2A2*) and sodium-dependent glucose cotransporters (*SGLT-1*) (47, 48) are listed in Table 4. *GAPDH* was applied as an internal control to normalize target gene expression levels.

**Table 4:** Primer sequences and target genes used for quantitative real-time PCR.

**Genes**	**Gene full name**	**Primer sequences (5^**′**^-3^**′**^)**	**Accession no**.
*AMY2A*	Pancreatic alpha 2A amylase	F-CGGAGTG^↓^GATGTTAACGACTGG R-ATGTTCGCAGACCCAGTCATTG	NM_001001473.2
*PNLIP*	Pancreatic lipase	F-GCATCTGGGAAG^↓^GAACTAGGG R-TGAACCACAAGCATAGCCCA	NM_001277382.1
*CCK*	Cholecystokinin	F-AGGTTCCACTGGGAGGTTCT R-CGCCTGCTGTTCTTTAGGAG	XM_015281332.1
*GLUT2*	Glucose transporter-2 (*SLC2A2*)	F-TGATCGTGGCACTGATGGTT R-CCACCAGGAAGAC^↓^GGAGATA	NM_207178.1
*SGLT-1*	Sodium-dependent glucose cotransporters	F-TGCCGGAGTATCTGAGGAAG R-CCCCATGGCCAACTGTATAA	XM_015275173.2
*GAPDH*	Glyceraldahyde-3-phosphate dehydrogenase	F-GGTGGTGCTAAGCGTGTTA R-CCCTCCACAATGCCAA	NM205518

### Partial Budget Analysis

Partial budget analysis of broiler production was estimated using economic analysis and profitability ratios. The partial budget analysis was performed to evaluate the economic advantage of the different treatments of olive pomace. Economic analysis was involved in the calculation of the feed costs and returns. The feed costs (average variable costs) were calculated by multiplying the actual feed intake for the whole feeding period with the prevailing prices. The net profit (NP) was calculated by subtracting total costs (TC) from total returns (TR). Feed costs/kg weight gain = FCR × cost of 1 kg diet (2).

Profitability ratios were used to explain how much of the factors of production were used for profit maximization and were calculated according to Verspecht et al. (49).

Benefit–cost ratio (BCR) = TR/TC.

Rate of returns on investment (RRI, %) = NP/TC × 100.

Profitability index (PI) = NP/TR.

Economic efficiency (EE) = NP/feed costs.

### Statistical Analyses

All statistical analyses were subjected to the GLM procedure of SPSS. Homogeneity and normality among the experimental groups were tested by Levene's and Shapiro–Wilk's tests, respectively. Tukey's *post-hoc* was done to test the significant differences among the mean values. Variation in the data was expressed as the standard error of the mean (SEM) and the significance was set at *p* < 0.05. Relative fold changes in the expression of target genes were calculated by the 2^−Δ*ΔCt*^ method according to Livak and Schmittgen (50).

## Results

### Growth Performance Indices

Growth performance data are presented in Table 5. Dietary inclusion of FOPII at the levels of 7.5 and 15% showed the highest body weight, weight gain, and feed intake (*p* < 0.05) during the start period. Moreover, FCR was improved (*p* < 0.05) in groups fed FOPII diets. Birds fed FOPII at the level of 15% had higher body weight, weight gain, and better FCR when compared with the control group during the grower–finisher period (*p* < 0.05). During the whole growing period (days 1–42), body weight and weight gain were significantly increased (*p* < 0.05) in groups fed FOPII (7.5, 15, and 20%) followed by group fed FOPI at the level of 7.5% as compared with the control group. The groups fed FOPII at the levels of 7.5 and 15% displayed the best FCR and PER.

**Table 5:** Effect of fermented or enzymatically fermented dried olive pomace broiler chickens' diet on growth performance parameters (starter, grower–finisher period, and overall period).

	**Control**	**FOPI 7.5%**	**FOPI 15%**	**FEOPI 30%**	**FOPII 7.5%**	**FOPII 15%**	**FOPII 30%**	**SEM**	***p*-value**
**Starter period (1–21 days)**									
Initial body weight	47	45	46	46	44	45	47	0.63	0.08
Body weight, g/bird	995^e^	1,017^c^	966^f^	872^g^	1,120^b^	1,208^a^	1,005^d^	14.42	<0.001
Body weight gain, g/bird	948^d^	972^c^	919^d^	826^d^	1,075^b^	1,163^a^	959^d^	13.54	<0.001
Feed conversion ratio	1.31^a^	1.32^a^	1.33^a^	1.48	1.26^b^	1.18^c^	1.21^b^	0.01	<0.001
Feed intake, g/bird	1,238^d^	1,282^c^	1,231^e^	1,223^f^	1,358^b^	1,368^a^	1,169^g^	9.75	<0.001
**Grower–finisher period (22–42 days)**									
Body weight, g/bird	2,485^d^	2,529^c^	2,378^e^	2,230^f^	2,603^b^	2,747^a^	2,475^d^	22.00	<0.001
Body weight gain, g/bird	1,490^b^	1,512^ab^	1,412^c^	1,358^d^	1,484^b^	1,539.4^a^	1,470^b^	8.87	<0.001
Feed conversion ratio	1.90^b^	1.88^bc^	2.00^a^	2.08^a^	1.85^c^	1.84^c^	1.93^b^	0.01	<0.001
Feed intake, g/bird	2,837^a^	2,841^a^	2,820^ab^	2,828^ab^	2,754^b^	2,829^ab^	2,836^a^	6.51	0.027
**Total growing period (1–42 days)**									
Feed intake, g/bird	4,074^b^	4,124^bc^	4,051^bc^	4,052^bc^	4,111^b^	4,196^a^	4,005^c^	9.78	<0.001
Body weight gain, g/bird	2,438^d^	2,484^f^	2,332^e^	2,184^c^	2,559^b^	2,702^a^	2,428^d^	20.08	<0.001
feed conversion ratio	1.67^c^	1.65^c^	1.74^b^	1.85^a^	1.61^d^	1.55^e^	1.65^a^	0.01	<0.001
Protein efficiency ratio	2.86^c^	2.88^c^	2.75^d^	2.58^e^	2.97^b^	3.07^a^	2.90^c^	0.02	<0.001

*Means within the same column carrying different superscripts (^a−g^) are significantly different at p < 0.05*.

*FOPI7.5%, 7.5% olive pomace subjected to microbial fermentation; FOPI15%, 15% olive pomace subjected to microbial fermentation; FOPI30%, 30% olive pomace subjected to microbial fermentation; FOPII7.5%, 7.5% olive pomace subjected to microbial and enzymatic fermentation; FOPII15%, 15% olive pomace subjected to microbial and enzymatic fermentation; FOPII30%, 30% olive pomace subjected to microbial and enzymatic fermentation*.

### Nutrient Digestibility

The digestibility coefficients of dry matter, crude protein, crude fiber, and ether extract are presented in Table 6. The highest dry matter digestibility was found in the group fed 15% of FOPII; in contrast, the group fed 30% FOP had the lowest dry matter digestibility. Groups fed FOPII at the levels of 7.5 and 15% showed higher (*p* < 0.05) crude protein and crude fiber digestibility when compared with the control group.

**Table 6:** Effect of fermented or enzymatically fermented dried olive pomace broiler chickens' diet on nutrient digestibility and carcass characteristics at slaughter (day 42).

**Parameters**	**Control**	**FOPI 7.5%**	**FOPI 15%**	**FEOPI 30%**	**FOPII 7.5%**	**FOPII 15%**	**FOPII 30%**	**SEM**	***p*-value**
**Nutrient digestibility, %**									
Dry matter	71.56^b^	71.24^b^	71.36^b^	68.96^c^	71.71^b^	73.02^a^	69.46^c^	0.17	<0.001
Crude protein	62.78^cd^	63.25^c^	63.01^c^	60.94^e^	65.05^b^	67.21^a^	61.91^de^	0.09	<0.001
Crude fiber	26.18^b^	25.73^b^	25.86^b^	24.13^c^	26.27^a^	27.28^a^	25.62^b^	0.04	<0.001
Ether extract	72.86^a^	69.98^b^	70.12^b^	72.60^a^	69.38^b^	70.30^b^	72.50^a^	0.06	0.04
**Chemical composition of meat**									
Breast moisture, %	72.60	72.80	72.86	72.63	72.64	72.86	72.5	0.007	0.052
Breast protein, %	22.82^b^	23.59^a^	22.94^ab^	23.00^ab^	23.56^a^	23.62^a^	23.14^ab^	0.09	0.008
Breast fat, %	3.28^a^	2.72^b^	2.46^bc^	2.34^c^	2.76^b^	2.38^c^	2.32^c^	0.005	<0.001
Cholesterol breast, mg/100 mg	63.26^a^	63.17^a^	61.93^b^	62.02^b^	62.62^ab^	62.01^b^	61.60^b^	0.005	0.002

*Means within the same column carrying different superscripts (^a−e^) are significantly different at p < 0.05*.

*FOPI7.5%, 7.5% olive pomace subjected to microbial fermentation; FOPI15%, 15% olive pomace subjected to microbial fermentation; FOPI30%, 30% olive pomace subjected to microbial fermentation; FOPII7.5%, 7.5% olive pomace subjected to microbial and enzymatic fermentation; FOPII15%, 15% olive pomace subjected to microbial and enzymatic fermentation; FOPII30%, 30% olive pomace subjected to microbial and enzymatic fermentation*.

### Expression of Glucose Transporter and Pancreatic Digestive Enzyme Genes

**Figure 1:**
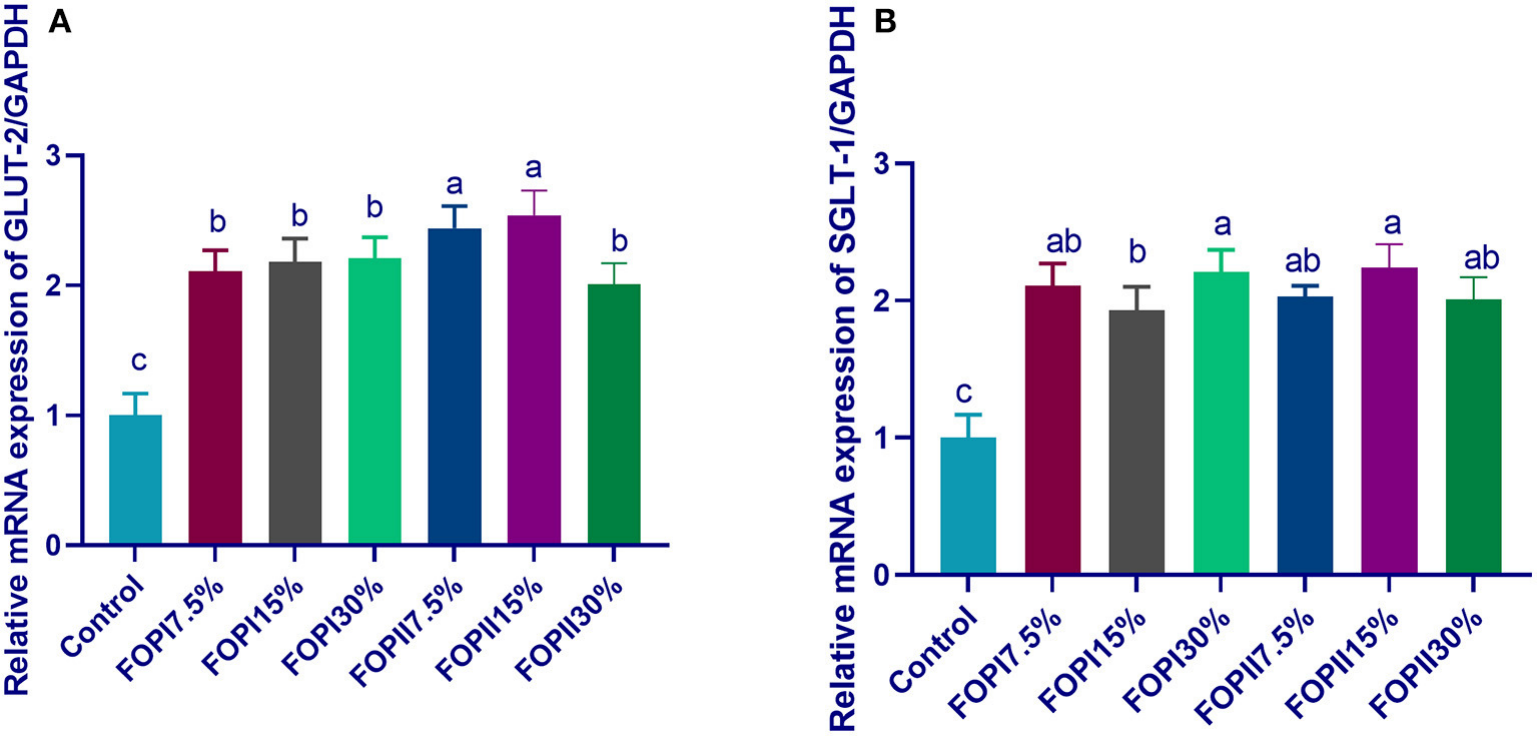
Effect of fermented or enzymatically fermented dried olive pomace on the expression of glucose transporter-2 [**(A)**; *GLUT2*] and sodium-dependent glucose cotransporters [**(B)**; *SGLT-1*]. FOPI7.5%, 7.5% fermented olive pomace; FOPI15%, 15% fermented olive pomace; FOPI30%, 30% fermented olive pomace; FOPII7.5%, 7.5% olive pomace subjected to microbial and enzymatic fermentation; FOPII15%, 15% olive pomace subjected to microbial and enzymatic fermentation; FOPII30%, 30% olive pomace subjected to microbial and enzymatic fermentation. Means within the same column carrying different superscripts (^a−*c*^) are significantly different at *p* < 0.05.

The mRNA expressions of the glucose transporter genes *GLUT2* and *SGLT*-1 are shown in Figure 1. Feeding on FOPI significantly upregulated (*p* < 0.05) the expression of GLUT2 and *SGLT-1*. With increasing level of FOPI in the diet of broilers, the expressions of *GLUT2* and *SGLT*-1 were significantly upregulated (*p* < 0.05). The maximum upregulation of *GLUT2* and *SGLT*-1 was observed in the 7.5% FOPII and 15% FOPII groups where external enzymes are used during the fermentation process. The mRNA expression of *SGLT-1* was significantly upregulated in groups fed FOPI and FOPII at the levels of 15 and 30%.

**Figure 2:**
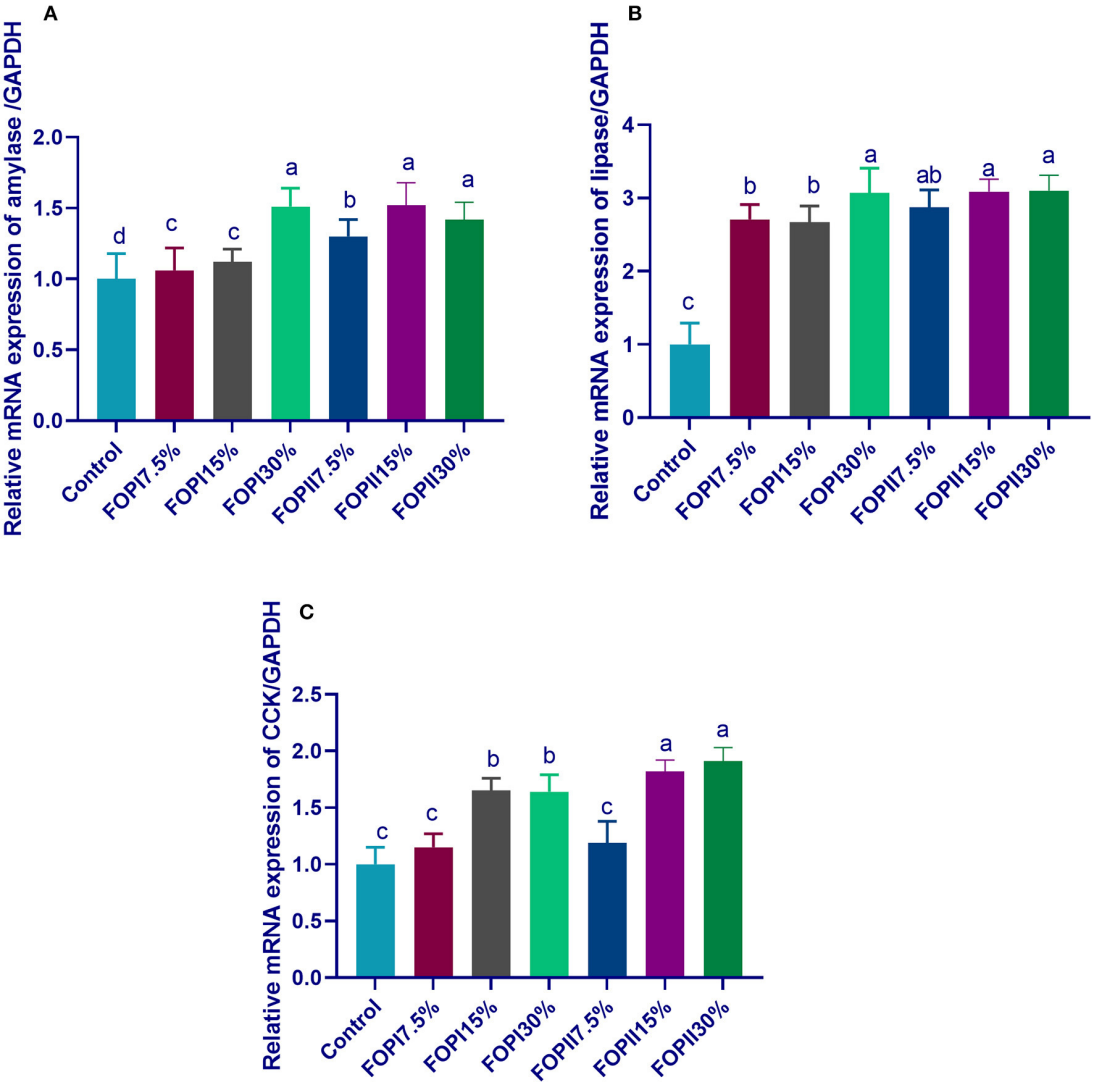
Effect of fermented or enzymatically fermented dried olive pomace on the expression of pancreatic alpha 2A amylase [**(A)**; *AMY2A*], lipase [**(B)**; *PNLIP*], and cholecystokinin [**(C)**; *CCK*]. FOPI7.5%, 7.5% fermented olive pomace; FOPI15%, 15% fermented olive pomace; FOPI30%, 30% fermented olive pomace; FOPII7.5%, 7.5% olive pomace subjected to microbial and enzymatic fermentation; FOPII15%, 15% olive pomace subjected to microbial and enzymatic fermentation; FOPII30%, 30% olive pomace subjected to microbial and enzymatic fermentation. Means within the same column carrying different superscripts (^a−*d*^) are significantly different at *p* < 0.05.

The expression of *AMY2A* was significantly upregulated in groups fed 20% FOPI and FOPII (15 and 30%) (Figure 2). The expression of lipase gene was significantly upregulated after feeding on FOPI and FOPII, while maximum upregulation was observed in groups fed 20% FOPI and 15 and 20% FOPII. The groups fed 15 and 30% of FOPI and FOPII showed upregulated levels of the *CCK* gene. The action of external fibrolyitc enzymes as additives during the solid-state fermentation of olive pomace was more prominent in upregulating pancreatic *AMY2A, PNLIP*, and *CCK* genes.

### Chemical Composition of Breast Meat

The effects of fermented olive pomace with and without enzyme on the chemical composition of meat are shown in Table 6. The percentage of breast protein was significantly increased in groups fed 7.5% FOPI and 7.5 and 15% FOPII when compared with the other groups. No significant differences were observed in moisture percentage among the experimental groups. The lowest fat % and cholesterol content (*p* < 0.05) in breast meat were observed in groups fed higher levels of FOPI and FOPII.

### Blood Biochemical Parameters

Serum biochemical parameters of broilers supplemented with FOPI or FOPII are shown in Table 7. No significant differences were observed in the serum concentration of ALT, AST, and LDL-C among the experimental groups. Broilers fed 15 and 30% FOPI and FOPII had low serum TG levels compared with groups fed 7.5% FOPI and FOPII and the control group (*p* < 0.05). All experimental groups, except the 7.5% FOPI group, exhibited lower serum cholesterol concentrations than the control group.

**Table 7:** Effect of fermented or enzymatically fermented dried olive pomace broiler chickens' diet on serum biochemical parameters at slaughter (day 42).

**Parameters**	**Control**	**FOPI 7.5%**	**FOPI 15%**	**FEOPI 30%**	**FOPII 7.5%**	**FOPII 15%**	**FOPII 30%**	**SEM**	***p*-value**
ALT, U/L	1.49	1.55	1.52	1.63	1.51	1.56	1.56	0.09	0.33
AST, U/L	48.63	49.05	49.04	48.58	48.4	49.08	50.08	0.19	0.27
Triglycerides, mg/dl	27.90^a^	28.16^a^	24.56^bc^	23.91^c^	28.40^a^	26.60^ab^	25.24^b^	0.30	<0.001
Total cholesterol, mg/dl	132.24^a^	132.00^a^	124.82^bc^	123.48^bc^	126.18^b^	122.68^c^	112.98^d^	0.42	<0.001
LDL-C, mg/dl	94.16	93.72	93.40	93.92	94.00	93.10	94.12	0.58	0.332

*Means within the same column carrying different superscripts (^a−d^) are significantly different at p < 0.05*.

*FOPI7.5%, 7.5% olive pomace subjected to microbial fermentation; FOPI15%, 15% olive pomace subjected to microbial fermentation; FOPI30%, 30% olive pomace subjected to microbial fermentation; FOPII7.5%, 7.5% olive pomace subjected to microbial and enzymatic fermentation; FOPII15%, 15% olive pomace subjected to microbial and enzymatic fermentation; FOPII30%, 30% olive pomace subjected to microbial and enzymatic fermentation*.

### Meat Oxidative Stability

Antioxidant and lipid oxidation biomarkers in relation to different levels of FOPI or FOPII are presented in Table 8. In serum and breast meat, the activity of T-AOC was increased with increasing the inclusion levels of FOPI or FOPII. However, no significant differences were observed among the groups fed FOPI and FOPII at the level of 7.5% and the control diet. Additionally, the activities of total SOD and GSH-Px were positively correlated to dietary inclusion levels of FOPI and FOPII. The highest GSH-Px activity was observed in groups fed FOPI and FOPII (15 and 30%).

**Table 8:** Effect of fermented or enzymatically fermented dried olive pomace broiler chickens' diet on antioxidant markers in serum and breast meat.

**Parameters**	**Control**	**FOPI 7.5%**	**FOPI 15%**	**FEOPI 30%**	**FOPII 7.5%**	**FOPII 15%**	**FOPII 30%**	**SEM**	***p*-value**
**Serum**									
T-AOC (U/mg of protein)	17.92^d^	18.07^d^	21.56^c^	22.94^ab^	17.92^d^	23.38^a^	21.84^bc^	0.41	<0.001
T-SOD (U/mg of protein)	15.338^d^	17.44^c^	18.34^b^	18.74^a^	17.68^c^	18.60^ab^	18.72^a^	0.20	<0.001
GSH-Px (AU)	281.86^d^	287.06^c^	295.06^b^	292.46^b^	286.04^c^	299.08^a^	294.52^b^	0.99	<s0.001
**Breast meat**									
T-AOC (U/mg of protein)	8.88^d^	9.26^cd^	13.26^b^	13.50^ab^	9.82^c^	13.82^ab^	14.02^a^	0.38	<0.001
GSH-Px (AU)	301.68^c^	301.78^c^	329.24^a^	325.60^b^	296.60^cd^	328.22^ab^	328.08^ab^	0.05	<0.001
Total-SOD (U/mg of protein)	26.34^d^	27.40^c^	30.98^a^	30.66^ab^	27.84^c^	30.23^b^	31.06^a^	2.39	<0.001
Total phenolic compounds (μg/g) at day 90	96.23^c^	119.30^b^	136.50^a^	138.3^a^	121.18^b^	135.64^a^	140.86^a^	2.23	<0.001
Total phenolic compounds (μg/g) at day 180	92.70^c^	116.28^b^	125.9^a^	127.32^a^	119.86^b^	128.76^a^	130.72^a^	0.63	<0.001
Total flavonoids (μg/g) at day 90	120.30^b^	121.20^b^	132.52^a^	133.42^a^	121.86^b^	134.04^a^	134.38^a^	1.78	<0.001
Total flavonoids (μg/g) at day 180	101.10^c^	118.50^b^	132.20^a^	132.84^a^	121.50^b^	129.88^a^	132.76^a^	0.63	<0.001
MDA (mg gallic acid/100 g meat) at day 90	0.15^a^	0.15^a^	0.11^bc^	0.09^c^	0.148^a^	0.092^c^	0.12^b^	0.01	<0.001
MDA (mg gallic acid/100 g meat) at day 180	0.76^a^	0.63^b^	0.55^c^	0.45^d^	0.67^b^	0.56^c^	0.43^d^	0.19	<0.001
DPPH (μM/g) at day 90	5.18^c^	5.4^c^	6.68^b^	6.80^b^	5.32^c^	7.14^a^	6.55^b^	0.03	<0.001
DPPH (μM/g) at day 180	3.43^b^	4.10^b^	6.37^a^	6.98^a^	4.12^b^	6.68^a^	6.43^a^	0.21	<0.001

*T-AOC, total antioxidative capacity; T-SOD, total superoxide dismutase; GSH-Px, glutathione peroxidase; MDA, malondialdehyde; DPPH, 1,1-diphenyl-2-picrylhydrazyl radical; TPC, total phenolic contents (μg/g); FOPI7.5%, 7.5% olive pomace subjected to microbial fermentation; FOPI15%, 15% olive pomace subjected to microbial fermentation; FOPI30%, 30% olive pomace subjected to microbial fermentation; FOPII7.5%, 7.5% olive pomace subjected to microbial and enzymatic fermentation; FOPII15%, 15% olive pomace subjected to microbial and enzymatic fermentation; FOPII30%, 30% olive pomace subjected to microbial and enzymatic fermentation*.

*Means within the same column carrying different superscripts (^a−d^) are significantly different at p < 0.05*.

In breast meat samples, total phenolic and flavonoid contents were varied among the different groups. After 90 and 180 days of frozen storage period, the highest TPC and TFC were detected in the groups fed higher levels of FOPI and FOPII (15 and 30%). The MDA values were significantly decreased in all the groups fed higher levels of FOPI or FOPII when compared with the control group (*p* < 0.05). Moreover, the content of MDA in breast meat significantly increased (*p* < 0.05) after frozen storage, but their values remained low in groups fed increased levels of FOPI and FOPII (15 and 30%) when compared with the control group. Compared with the control group, inclusion of fermented olive pomace at high levels significantly increased (*p* < 0.05) the ability of meat to scavenge free radical DPPH, and this capacity decreased with the storage period. Meanwhile, the free radical scavenging activity of DPPH remained higher in groups fed higher levels of fermented olive pomace in comparison with the control group.

### Budget Analysis

Partial budget analysis (economic analysis and profitability ratios) of the different dietary treatments of fermented olive pomace with or without enzymes is presented in Table 9. The highest significant feed and total costs were found in the control group and the 7.5 and 15% FOPII-substituted groups. Meanwhile, the lowest feed and total costs were found in groups fed 15 and 30% FOPI and FOPII. TR and NP were significantly increased (*p* < 0.05) in groups fed 15 and 7.5% FOPII compared with the other treated groups. The lowest cost/kg body weight of birds was found in groups fed 7.5 and 15% FOPII, while the highest cost/kg was found in groups fed 15 and 20% FOPI. The profitability ratios (BCR, PI, RRI, and EE) were significantly increased in groups fed 15% FOPII followed by groups fed 7.5% FOPII. Moreover, the group substituted with 30% FOPI achieved the lowest profitability ratios.

**Table 9:** Effect of fermented or enzymatically fermented dried olive pomace broiler chickens' diet on economics data.

**Treatments**	**Control**	**FOPI 7.5%**	**FOPI 15%**	**FEOPI 30%**	**FOPII 7.5%**	**FOPII 15%**	**FOPII 30%**	**SEM**	***p*-value**
Feed cost, $	1.71^a^	1.68^b^	1.59^c^	1.55^d^	1.70^ab^	1.68^ab^	1.58^c^	0.002	<0.001
TC, $	2.68^a^	2.65^b^	2.56^c^	2.516^d^	2.670^ab^	2.65^ab^	2.55^c^	0.001	<0.001
TR, $	3.92^d^	3.99^c^	3.75^e^	3.52^f^	4.11^b^	4.34^a^	3.91^d^	0.01	<0.001
NP, $	1.24^d^	1.34^c^	1.19^d^	1.00^e^	1.44^b^	1.68^a^	1.36^c^	0.004	<0.001
Feed cost/kg gain, $	0.71^a^	0.68^bc^	0.68^b^	0.68^b^	0.67^c^	0.62^d^	0.66^c^	0.001	<0.001
BCR	1.46^d^	1.51^c^	1.47^d^	1.40^e^	1.54^b^	1.64^a^	1.53^b^	0.003	<0.001
PI	0.32^d^	0.34^c^	0.32^d^	0.29^e^	0.35^b^	0.39^a^	0.35^bc^	0.001	<0.001
RRI, %	46.25^d^	50.59^c^	46.48^d^	39.90^e^	53.93^b^	63.34^a^	53.35^b^	0.01	<0.001
EE%	72.42^d^	79.71^c^	74.74^d^	64.86^e^	84.60^b^	99.82^a^	86.02^b^	0.04	<0.001

*TC, total costs; TR, total returns; NP, net profit; BCR, benefit–cost ratio; PI, profitability index; RRI, rate of returns on investment; EE, economic efficiency; FOPI7.5%, 7.5% fermented olive pomace; FOPI15%, 15% fermented olive pomace; FOPI30%, 30% fermented olive pomace; FOPII7.5%, 7.5% olive pomace subjected to microbial and enzymatic fermentation; FOPII15, 15% olive pomace subjected to microbial and enzymatic fermentation; FOPII30%, 30% olive pomace subjected to microbial and enzymatic fermentation*.

*Price of kg feed = control (0.420$), FOPI7.5% (0.408$), FOPI15% (0.393$), FOPI30% (0.382$), FOPII7.5% (0.414$), FOPII15% (0.401$), FOPII30% (0.394$). AFC of all groups = 0.968$ and selling costs of 1 kg meat = 1.58$*.

*Means within the same column carrying different superscripts (^a−f^) are significantly different at p < 0.05*.

## Discussion

Food industries yield a large quantity of DOP, which has been successfully used in animal nutrition. Dried olive pomace is considered as a complementary energy source; besides, it represents an important source of bioactive compounds such as polyphenols with high free radical scavenging capacity (51, 52). However, the utilization of DOP by broiler chickens may be hindered due to its higher crude fiber content. Thus, microbial fermentation with added exogenous enzymes can be used as an effective approach to improve the nutritional quality of DOP. Our study cleared that the chemical composition of OP was changed after microbial fermentation. Moreover, the addition of external fibrolytic enzymes during the fermentation process accounted for more biodegradation of crude fiber content in OP that resulted in an additional decrease in crude fiber content and an increase of crude protein content. These results were supported by the findings of Fathy et al. (7), who described that solid-state fermentation improved the chemical composition of olive pulp, so that the contents of crude protein, fat, and carbohydrate were increased by 2.74, 2.63, and 3.57%, respectively, while crude fiber content was reduced by 8.56%. Similarly, microbial fermentation of rapeseed meal increased the crude protein content from 387 to 423 g/kg (53). Moreover, a better quality of OP was found in FOPII where external fibrolytic enzymes were added. These exogenous enzymes can enhance the action of enzymes secreted during fermentation and resulted in more biodegradation of plant cell walls. Similarly, the addition of exogenous enzymes together with microbial inoculants has been shown to improve the fermentation of tropical grasses (54). Treatment of bermudagrass with an enzyme–inoculant combination (54, 55) or fibrolytic enzymes alone improved the fermentation process. Additionally, increasing lactic acid bacteria in FOPII could be due to the presence of more substrates of fermentable carbohydrates that activate lactic acid bacteria and stimulate a good fermentation process (56). Moreover, the addition of enzymes as additives during fermentation can enhance the breakdown of plant cell walls and increase the release of plant cell wall carbohydrates, thus providing sugars for the lactic acid bacteria that account for their activation (57). Moreover, reduction of fiber content and increased carbohydrate content are additional benefits from the biological treatment of grasses (58). Additionally, a higher concentration of phenolic compounds was detected after fermentation of OP, and this can be attributed to the breakdown of the plant cell wall and subsequent enzyme activities that lead to the release of bound phenolic compounds, which enhance the antioxidant activities of the fermented product (59). Also, enhancement of β-glucosidase activities during fermentation is responsible for hydrolyzing phenolic glycosides and releasing free phenolics during the fermentation of plants which account for their higher TPC contents (60). Furthermore, elevated TPC in fermented products can be explained by the higher metabolic activity of microbes that modify the levels of bioactive compounds (61). Enhancing the nutritive value of olive pomace by solid-state fermentation especially with the addition of fibrolytic enzymes is in accordance with enhancing growth performance parameters of broiler chickens. Better growth performance of broiler chickens in the current study was observed in groups fed FOPII up to 15% than groups fed FOPI, which may result from the improved nutritional properties of FOPII in these groups. These results are supported by Uchewa and Onu (62) who stated that feeding fermented feed (Bactocell as starter inoculum) improved the feed intake and weight gain of broiler chickens. Fermented feeds can be responsible for improving digestion and absorption which in turn enhance the growth performance of birds (25, 31, 63). Additionally, microbial fermentation has a positive impact on gut health and growth performance of broiler chickens (34, 64). Likewise, the inclusion of an enzyme blend (glucanase, pectinase, and xylanase) during fermentation of rapeseed cakes was highly effective in lowering their non-starch polysaccharides (NSP) by 31 to 42% (65). Furthermore, fermentation of an ingredient could offer a high number of beneficial microorganisms with probiotic effects on the GIT (31). Moreover, the application of microbial strains during fermentation of olive pomace in our study not only improves its nutritional value but also affects broilers' performance. In agreement with our results, microbial fermentation with *B. subtilis* can increase feed palatability (66), secrete digestive enzymes (proteases, amylases, and lipases) to decompose plant complex carbohydrates, and promote nutrient digestion and absorption. Additionally, it generates active compounds (bacitracin, nystatin, polymyxin, and gramicidin) inhibiting the effect of endogenous pathogens (67). Moreover, the addition of *Saccharomyces cerevisiae* is preferred during anaerobic fermentation process due to its greater acidic capacity (68) as well as its enzyme-producing ability such as β-glucanase, phytase, and invertase (69). The better performance of broiler chicks in groups fed FOPII was higher than the other groups, which may be attributed to the presence of functional metabolites resulting from microbial fermentation. Moreover, the addition of fibrolytic exogenous enzymes augments the action of microbial enzymes secreted during the fermentation process.

On the other hand, increasing the nutrient digestibility of dry matter, crude protein, and crude fiber in the group fed 15% FOPII is in accordance with Dean et al. (55), who stated that cellulase, xylanase preparations can hydrolyze the cell walls in the grasses into sugars, which stimulated homolactic bacteria growth and resulted in an increase in lactic acid concentration and DM, NDF, and ADF digestibility of the grass. Also, crude fiber reduction indicated an improved digestibility of the resultant substrate (70). Fermentation can improve the nutritive value and digestibility of unconventional feed ingredients (30). Moreover, the fermented feed has positive impacts in the gastrointestinal tract such as a drop in gastric pH and a decrease of pathogenic microbial activity along with an increase of short-chain fatty acid production, which could result in better growth performance of the chickens (71). Moreover, enhancing digestive enzyme activities (amylases, lipases, proteases, and trypsin) after feeding on fermented feeds is accountable for improving the growth rate of the birds (72, 73). Supplementation of exogenous enzymes decomposes the NSP and decreases intestinal viscosity, which consequently lead to better nutrient digestibility and growth performance of birds (74). The positive effects of the enzymes can be attributed to the interruption of the plant cell wall integrity and subsequent release of encapsulated nutrients (28). Digestive enzymes play a critical role in decomposing the feed particles and releasing nutrients for absorption, which are essential for the growth and general health of the birds. Enhancing the expression of genes encoding digestive enzymes can increase digestive enzyme activities and feed utilization in broiler chickens (75). Upregulation of genes encoding pancreatic enzymes in groups fed fermented olive pomace especially with enzymatic treatment was observed in this study. In a similar manner, Al-Khalaifah et al. (2) stated that feeding on microbially fermented dried brewers grains upregulated the expressions of pancreatic enzyme genes (amylase, lipase, and protease) in broiler chickens. Additionally, the activities of pancreatic enzymes in broilers were enhanced after feeding fermented soybean meal (76). To the best of our knowledge, data reporting the underlying mechanisms by which fermented feeds may enhance digestive enzymes activities are limited. Indirectly, reducing the load of pathogenic bacteria resulting from feeding good quality fermented feed may augment digestive enzyme secretion. Fermented feeds can also lower pH in the upper GI tract and create an unfavorable environment for colonization and growth of pathogenic bacteria which may inhibit the secretion of digestive enzymes by damaging the intestinal mucosa of chickens (77). Furthermore, the activities of protease and amylase enzymes in broilers were enhanced after feeding on cottonseed meal fermented with *B. subtilis* (73) that resulted from *B. subtilis* contribution in the protease and amylase enzyme production. Additionally, increasing carbohydrate intake can increase the expression levels of mRNA of glucose transporters which increase glucose absorption (78). In our study, *GLUT2* and *SGLT1* were upregulated in groups fed different levels of FOPI or FOPII. Similarly, adding exogenous dietary enzymes for broilers increased the intestinal *GLUT2* expression and facilitated micronutrient absorption (79). Also, the expression of the *GLUT2* gene was upregulated after 2 and 3 weeks of xylanase inclusion, which may suggest an increased absorption in birds (80). Moreover, OP contains several plant metabolites, especially phenolic and flavonoid compounds. The expressions of *GLUT2* and *SGLT1* genes may also be affected by plant bioactive compounds present in the OP (81, 82). The addition of a mixture of carbohydrate-based enzymes to the wheat and barley diets significantly decreased the digesta viscosity and elevated the activities of α-amylase and lipase (83). In Arbor Acres broilers, amylase activity was increased with *Bacillus coagulants* supplementation (32). Also, upregulation of mRNA expression of pancreatic lipase in all groups fed either FOPI or FOPII may elevate the lipase activity and induce effective fat absorption (84). Also, Lee et al. (85) described that *B. subtilis*-based direct-fed microbials upregulated the expressions of pancreatic lipase, carboxypeptidase, and chymotrypsin-like elastase family genes in the gut. In our study, the application of probiotics bacteria (*B. subtilis*) during fermentation of olive pomace can lead to an improvement in brush border enzyme expression leading to better digestion and absorption. Moreover, increasing levels of FOPII (15 and 30%) had a more prominent effect of decreasing cholesterol and fat content of breast meat. Similarly, Kovalík et al. (86) stated that feeding on 5% fermented feed resulted in a lower percentage of breast muscle fat. Furthermore, dietary inclusion of olive cake at the level of 57 g/kg decreases cholesterol and total saturated fatty acid levels and increases total monounsaturated fatty acid, total n-3 PUFA, and docosahexaenoic acid in egg yolk (87). Fat and saturated fatty acid contents in pig meat were reduced after feeding on olive cake (88). Broilers fed fungal solid-state fermented products showed a lower meat fat content (89). Decreasing of cholesterol content may be raised from the products of fermentation such as short-chain fatty acids that suppress hepatic cholesterol synthesis (90) and stimulate bile acid synthesis (91). In addition, our study described that the protein content of breast meat was slightly increased in all groups fed fermented olive pomace. In a similar manner, Hossain and Yang (92) described that feeding 0.5% fermented water plantain increased breast protein content and decreased thigh fat content of broiler chickens. Moreover, elevated crude protein and reduced crude fat contents in breast meat of broilers were detected after feeding on microbially fermented *Alisma canaliculatum* (93, 94). Also, feeding of broilers on 5 and 10% fungal solid-state fermented feed lowered fat percentage and increased protein percentage in breast meat (89). Increasing breast protein content in groups fed 7.5% FOPI and 7.5 and 15% FOPII may be attributed to increased availability of fermented bioactive compounds in these groups, which in turn promote protein synthesis in meat (95). Moreover, feeding on fermented feed significantly increased lean meat (low fat and high protein content) in pigs that can be explained by increased protein synthesis signaling and suppressed degradation signaling in skeletal muscle through regulation of insulin signaling pathway (96). Antioxidant capacity is important for animal health, and it is one of the most crucial factors influencing meat quality after slaughter (97). Lipid peroxidation triggered by higher levels of free radicals not only induces oxidative stress and increases the content of MDA (98), but also causes the deterioration of meat. So, improving the antioxidant properties of meat is of great significance to improve meat quality and prolong meat storage time. Similarly, Gao et al. (99) indicated that the dietary inclusion of antioxidants can alleviate the adverse effects of oxidative stress on broiler production and improve meat quality. In the current study, the highest antioxidant capacity of serum and breast meat was found in groups fed higher levels of FOPI or FOPII. Decreased oxidation rates in meat after long storage (180 days of storage) were observed in groups fed higher levels of fermented olive pomace since it contains phenolic compounds with radical scavenging and antioxidant activities such as oleuropein, oleacein, oleocanthal, tyrosol, and hydroxytyrosol (100, 101). The protective effects of phenolics in biological systems are associated with their ability to transfer electrons, activate antioxidant enzymes, chelate metal, and reduce α-tocopherol radicals (102, 103). Likewise, improved oxidative stability was found after administration of higher levels of olive by-products into the broilers' (18, 104) and rabbits' diet (105). Also, pig meat oxidation stability assessed by MDA values tended to be improved in the DOP group after 1 day of refrigerated storage (106). Previous studies have examined the polyphenol content and the antioxidant capacity of several by-products and reported that the addition of by-products from olive mill wastewater processed using ceramic membrane microfiltration to the chicken diet improved their redox status (13). In the current study, diets with higher levels of fermented olive pomace showed more effective DPPH radical scavenging activities than the control diet. Similarly, fermented barley or wheat had more potent DPPH radical scavenging activities than the control diet (107). Moreover, the antioxidant function of fermented soybean meal might be derived from its fermentation products by reduced breast hydrogen peroxide level (108). It was described that during the microbial fermentation of soybean meal with *Bacillus amyloliquefaciens*, the antioxidant compounds, such as the phenolic and flavonoid compounds, increased sharply and the administration of lyophilized FSBM supernatant enhanced the T-SOD, glutathione peroxidase, catalase activities, and total antioxidant capacity and inhibited MDA formation in the serum and liver of mice (109). In broilers' diets, replacing corn with 10% fermented wheat bran increased the expression of antioxidant genes (110). Dietary supplementation of solid-state fermented *Isaria cicadae* in broiler chickens' diet enhanced GSH-Px, T-AOC, and T-SOD activities owing to the presence of polyphenols scavenging free radicals (111). The success of a poultry flock can be measured by economic production assessment. Feed is a significant input that contributes ~70–80% of overall cost of rearing broilers. Moreover, fermentation is an economical means to improve the nutritional quality of novel unconventional feed ingredients and enhance immune function and growth performance of broiler chickens (34). Also, the addition of exogenous enzymes during fermentation could improve the production efficiency of poultry by increasing the digestion of low-quality products and reducing nutrient loss with likely economic advantages (112). The current study described that the additional costs of fermentation of olive pomace were low when compared with the price of conventional feed ingredients. Using 15% FOPII followed by 7.5% FOPII was economically much more profitable than the control diet as these groups provided better growth performance and earning profit. Similarly, economic evaluation of olive pulp inclusion for up to 15% in broilers' diet indicated that both cost and profit indices resulting from olive pulp inclusion were better than corn-based diets (15). Additionally, broilers fed 10% fermented dried brewer grains had higher economic efficiency compared with the control group (2). So, using low-cost economical feed such as fermented olive pomace for broilers could compensate for the negative effects of higher prices of a conventional diet.

## Conclusion

Fermentation of olive pomace especially in the presence of exogenous enzymes can enhance its nutritional quality and encourage its application as a substitute for higher-cost conventional feed ingredients in the poultry industry. Additionally, feeding of fermented olive pomace had a prominent role on nutrient digestibility and glucose absorption by augmenting the expression of digestive enzymes and glucose transporter genes. Among the fermented olive pomace-fed groups, the group fed 15% FOPII achieved the highest body gain (increased by 12.5% vs. control) and net profit (increased by 26% vs. control) with high-quality meat enriched with antioxidant compounds that can guarantee satisfactory oxidative stability in poultry meat offered for human consumers. Finally, from an economic point of view, 15% FOPII can be profitable and economically applicable in poultry production farms.

## Data Availability Statement

The raw data supporting the conclusions of this article will be made available by the authors, without undue reservation.

## Ethics Statement

The animal study was reviewed and approved by Institutional Animal Care and Use Committee at Zagazig University.

## Author Contributions

All authors shared in designing the study, methodology, data collection and analysis, statistical analysis, and writing of the manuscript.

## Conflict of Interest

The authors declare that the research was conducted in the absence of any commercial or financial relationships that could be construed as a potential conflict of interest.
